# Differences in health outcomes for high‐need high‐cost patients across high‐income countries

**DOI:** 10.1111/1475-6773.13735

**Published:** 2021-08-11

**Authors:** Irene Papanicolas, Kristen Riley, Olukorede Abiona, Mina Arvin, Femke Atsma, Enrique Bernal‐Delgado, Nicholas Bowden, Carl Rudolf Blankart, Sarah Deeny, Francisco Estupiñán‐Romero, Robin Gauld, Philip Haywood, Nils Janlov, Hannah Knight, Luca Lorenzoni, Alberto Marino, Zeynep Or, Anne Penneau, Andrew J. Schoenfeld, Kosta Shatrov, Mai Stafford, Onno van de Galien, Kees van Gool, Walter Wodchis, Ashish K. Jha, Jose F. Figueroa

**Affiliations:** ^1^ Department of Health Policy London School of Economics London UK; ^2^ Department of Health Policy and Management Harvard T.H. Chan School of Public Health Boston Massachusetts USA; ^3^ Centre for Health Economics Research and Evaluation (CHERE) University of Technology Sydney Australia; ^4^ Scientific Center for Quality of Healthcare Radboud University Medical Center, Radboud Institute for Health Sciences Nijmegen The Netherlands; ^5^ Institute for Health Sciences in Aragon (IACS) Zaragoza Aragon Spain; ^6^ Dunedin School of Medicine University of Otago Dunedin Otago New Zealand; ^7^ KPM Center for Public Management University of Bern Bern Switzerland; ^8^ Hamburg Center for Health Economics Universität Hamburg Hamburg Germany; ^9^ The Health Foundation London UK; ^10^ Otago Business School University of Otago Dunedin Otago New Zealand; ^11^ The Swedish Agency for Health and Care Services Analysis Stockholm Sweden; ^12^ Health Division Organisation for Economic Co‐operation and Development (OECD) Paris France; ^13^ Institute for Research and Documentation in Health Economics (IRDES) Paris France; ^14^ Department of Orthopedic Surgery Brigham and Women's Hospital Boston Massachusetts USA; ^15^ Swiss Institute for Translational and Entrepreneurial Medicine Bern Switzerland; ^16^ Zilveren Kruis Leusden The Netherlands; ^17^ Institute of Health Policy Management & Evaluation University of Toronto Toronto Canada; ^18^ Brown School of Public Health Providence Rhode Island USA

**Keywords:** health systems, mortality, readmissions

## Abstract

**Objective:**

This study explores variations in outcomes of care for two types of patient personas—an older frail person recovering from a hip fracture and a multimorbid older patient with congestive heart failure (CHF) and diabetes.

**Data Sources:**

We used individual‐level patient data from 11 health systems.

**Study Design:**

We compared inpatient mortality, mortality, and readmission rates at 30, 90, and 365 days. For the hip fracture persona, we also calculated time to surgery. Outcomes were standardized by age and sex.

**Data Collection/Extraction Methods:**

Data was compiled by the International Collaborative on Costs, Outcomes and Needs in Care across 11 countries for the years 2016–2017 (or nearest): Australia, Canada, England, France, Germany, the Netherlands, New Zealand, Spain, Sweden, Switzerland, and the United States.

**Principal Findings:**

The hip sample across ranged from 1859 patients in Aragon, Spain, to 42,849 in France. Mean age ranged from 81.2 in Switzerland to 84.7 in Australia, and the majority of hip patients across countries were female. The congestive heart failure (CHF) sample ranged from 742 patients in England to 21,803 in the United States. Mean age ranged from 77.2 in the United States to 80.3 in Sweden, and the majority of CHF patients were males. Average in‐hospital mortality across countries was 4.1%. for the hip persona and 6.3% for the CHF persona. At the year mark, the mean mortality across all countries was 25.3% for the hip persona and 32.7% for CHF persona. Across both patient types, England reported the highest mortality at 1 year followed by the United States. Readmission rates for all periods were higher for the CHF persona than the hip persona. At 30 days, the average readmission rate for the hip persona was 13.8% and 27.6% for the CHF persona.

**Conclusion:**

Across 11 countries, there are meaningful differences in health system outcomes for two types of patients.

**What is known on this topic**

- Patient outcomes such as mortality and readmissions are commonly used as measures of performance of heatlh systems.
- There are few sources of comparable mortality rates at different time intervals for high‐need, high‐cost populations across countries.
- There are limited international comparisons of hospital readmission rates across countries.

**What this study adds**

- There is large variability in mortality rates at all time intervals for an older person with frailty and for an older multimorbid person across 11 high‐income countries.
- England and the United States have the highest mortality at 1 year for an older person with a hip fracture and for an older multimorbid person with heart failure and diabetes.
- Readmissions across countries are variable and may be related to mortality rates.

## 1 INTRODUCTION

A fundamental goal of all health systems is improving the health of their populations through high‐quality care.^1^, ^2^ International comparisons of this objective provide policy makers with the opportunity to assess their health system's relative performance and, importantly, have tremendous power to motivate action.^2^ However, measuring these outcomes across countries involves distinct challenges, due to the cross‐national variation present in the factors that influence them, such as patient characteristics, health system structures, and the organizations involved in health care delivery.^3^, ^4^

Current sources of comparable health outcomes data across countries are limited, and even more so if one wants to explore outcomes that measure the contribution of the health system to health outcomes.^2^, ^3^ While there are a few databases that regularly collect and report population health outcomes, such as life expectancies and avoidable mortality, these data are aggregated across all populations and conditions. Therefore, it is challenging for policy makers to clearly tease out how much of this variation is due to differences in service delivery. For specific conditions, such as cancer, acute myocardial infarction (AMI), and stroke, some international data are available, such as 30‐day case‐fatality rates from the Organization for Economic Cooperation and Development (OECD) or cancer survival rates from Eurocare. Other measures of health care quality, such as readmission rates or mortality figures for specific conditions, are only available from comparative European projects,^5^, ^6^ giving us only rare glimpses into comparative assessments of health care. As a result, we have limited information on the extent to which differences in health care system delivery influences quality for short‐ and long‐term outcomes, particularly for high‐need high‐cost (HNHC) patients. For policy makers to be able to take action on comparative results, they need more information on cross‐national outcomes for specific patient types to be able to robustly compare like with like.

In an attempt to address this gap, the International Collaborative on Costs, Outcomes and Needs in Care (ICCONIC) explore cross‐national variations in outcomes of care for two types of HNHC patients—an older frail person, identified as an older adult (older than aged 65 years) recovering from a hip fracture and a multimorbid older patient, identified as an older adult (aged 65–90 years) with a diagnosis of CHF and diabetes—across 11 countries: Australia, Canada, England, France, Germany, the Netherlands, New Zealand, Spain, Sweden, Switzerland, and the United States. Using an approach similar to tracer conditions,^5^, ^6^, ^7^, ^8^, ^9^, ^10^ which builds on previous international work, we define and follow these two specific types of HNHC patients over the course of a year to examine variation in short‐ and long‐term outcomes, across their pathway of care. Our study focuses on the following three questions: (1) How do health outcomes, such as mortality and readmission rates, vary for two distinct HNHC populations across countries over the course of a year? (2) How does process quality, such as time to surgery for hip fracture vary across countries? (3) and finally, To what extent are differences in patient comorbidity and length of stay (LOS) across countries correlated with mortality for these HNHC personas?

## 2 DATA AND METHODS

### 2.1 Data

The ICCONIC research collaborative collected linked patient‐level data from 2016 to 2017 (or the closest year) from multiple care settings—spanning primary care, specialty services, acute hospital care, and postacute care—across 11 countries, namely, Australia, Canada, England, France, Germany, the Netherlands, New Zealand, Spain, Sweden, Switzerland, and the United States. Each country collected data on patient characteristics, comorbidities, utilization, spending, and outcomes for two prespecified patient cohorts that reflect two different phenotypes of HNHC patients, as defined by the National Academy of Medicine HN/HC Framework.^11^ We identified two specific patient populations who would belong to each of these two categories as follows: an older frail person, identified as an individual aged older than 65 years with a hospital admission for hip fracture (the hip fracture persona) and a complex, multimorbid patient, identified as an individual aged 65–90 admitted to hospital with a primary diagnosis of congestive heart failure (CHF) and diabetes as a comorbidity (the CHF with diabetes persona). For a detailed overview of the methodology employed to collect the data, see methodology paper from Figueroa et al.^12^

Most countries used secondary/administrative data sets as the main source of information to estimate expenditure profiles. These data sets are large, linked datasets, which are coded and available in an electronic format. Specific details of each dataset used can be found in Table S1. The representativeness of the population for each dataset is found in Table S2. Data in three countries—New Zealand, Sweden, and Switzerland—covered their entire population. Data in three other countries were regional samples—Australia (NSW), Canada (Ontario), and Spain (Aragon region). Data in the remaining five countries were large, regionally diverse samples, including in England, France, Germany, the Netherlands, and the United States. The degree to the proportion of patients covered in each dataset varied across countries, from 3% in Spain (Aragon) to 100% in New Zealand, Sweden, and Switzerland.

Identification of both personas required at least 2 years of patient‐level data. Across most countries, we used 2 years between 2015 and 2017, except in Australia (used 2012–2016) and England (2014–2016), which had smaller samples and so pooled more years of data (See Table S2).The first year of data was used to identify all relevant patients through an inpatient admission, either a hip fracture or a heart failure exacerbation. We then tracked patients for different time intervals into the second year to measure service use and outcomes. For almost all countries (except Spain and the Netherlands), we used International Classification of Diseases, 10th revision (ICD‐10) codes, as defined by the World Health Organization. For the hip fracture persona, the codes included S72.0, S72.1, and S72.2, which represent fractures of the upper femur. In Spain, ICD‐9‐CM codes were used for relevant diagnosis and a customized diagnostic codes were derived with input of clinical experts in the Netherlands, given that ICD codes were not available in their insurer data. Within this group, we then focused on the patients who received one of three procedures as follows: total hip replacement, partial hip replacement, or osteosynthesis—which include placement of a screw, plate, pin, or internal fixation. Each country used a clinical expert to identify the relevant procedure codes.

For the heart failure persona, we identified all patients hospitalized with a primary diagnosis of CHF (ICD‐10 code I50.x) or relevant codes in Spain (using ICD‐9 codes) and in the Netherlands. Given the lack of comprehensive longitudinal data across most countries, we were unable to know if the hospitalization was the first hospitalization related to heart failure or not. We then identified the subset of patients who at time of the first admission also had a diagnosis of diabetes, including ICD‐10 codes of E11.x, E12.x, E13.x, and E14.x.

### 2.2 Key outcomes of interest

The main outcomes of interest in this article are mortality and readmission rates, including in‐hospital mortality and all‐cause mortality rates or readmission rates at 30, 90, and 365 days. For the hip fracture persona, we also calculated “time to surgery,” as previously defined by the OECD (described in further detail below).^13^

In‐hospital mortality is calculated by determining the number of patients who died within the index hospital admission, at any point in time, over the total sample population. All‐cause mortality is calculated by determining the number of patients who die within 30, 90, and 365 days from the day of the index hospital admission, including if they died after being discharged from the hospital admission, over the total sample population. All‐cause readmission rates are calculated by determining the number of patients who are readmitted to the hospital, within 30, 90, and 365 days from the day of discharge from the index hospital admission, over the total sample population. All countries, but Switzerland, were able to provide estimates of overall mortality and readmission over the 30‐, 90‐, and 365‐day period. Two countries, France and the Netherlands, were not able to identify the exact date of death outside of the hospital in their data. France was only able to identify the quarter of death and, therefore, does not report 30‐day overall mortality and may have slightly upward‐biased estimates for the other mortality estimates, aside from in‐hospital mortality. In the Netherlands, deaths were registered on the last date of each month. Therefore, an additional time window of 15 days was used calculate mortality rates. This may have slightly attenuated mortality estimates, especially for shorter outcome intervals like in‐hospital mortality and 30‐day mortality.

For the hip fracture persona, we also examined the “time to surgery” during index hospitalization. In line with the OECD,^13^ we used the “same day,” “next day,” “third day” terminologies to classify the day of procedure, starting from the first day of hospitalization, and report what percentage out of the total number of patients received a procedure. If there are multiple types of procedures reported for one patient, we counted the days to the first procedure that patients received. Hospital transfers were linked and defined as one episode.

### 2.3 Analysis

All outcomes were stratified by sex and specific age groups, allowing for direct age–sex standardization to the data. This adjusts for differences in the age and sex structures of the cohort. In total, we collected five groups per sex, per country for the CHF with diabetes persona (65–69, 70–74, 75–79, 80–84, 85–90 years, and last age category includes 6 years) and seven groups, per sex, per country for the hip fracture persona (65–69, 70–74, 75–79, 80–84, 85–90, 90–94, and 95 years or older as last group). As expected, given the specific nature of the pathways we are exploring, the sample populations differ significantly from the structure of the general population. For this reason, we used the US sample as the reference population rather than relying on an external standard.

While we collected comorbidity data for the aggregate groups of patients, we did not adjust for this, given the variability in coding systems across countries.^12^ To illustrate the magnitude of these coding differences, we used scatterplots to depict the association of 30‐day mortality and number of comorbidities by age–sex groupings, across countries (seven groups for the hip persona and five groups for the CHF persona). In addition, we used scatter plots to examine the association between inpatient morality and index LOS. We also examined the association between 30‐day mortality and 30‐day readmission rates, which are competing outcomes.

## 3 RESULTS

### 3.1 Patient characteristics across countries

For the hip persona, sample sizes ranged from 1859 in Aragon, Spain, to 42,849 patients in the France (See Table S1). Mean age (standard deviation [SD]) ranged from 81.2 (6.9) in Switzerland to 84.7 (7.7) in Australia. The majority of the hip fracture samples across countries were female, ranging from 62.8% in Australia to 76.7% in Spain. Germany and the United States had the highest mean number of comorbidities recorded in their datasets relative to other countries.

Sample sizes for the heart failure persona with diabetes ranged from 742 in England to 21,803 in the United States. Mean age (SD) ranged from 77.2 (7.0) in the United States to 80.3 (6.8) in Sweden. Across most countries, except in the United States, there were more males than females represented in their samples. The United States and Germany recorded the highest number of comorbidities per person as compared to other countries. Sweden and Canada recorded the fewest number of comorbidities for both personas.

### 3.2 Mortality rates across countries

**FIGURE 1:**
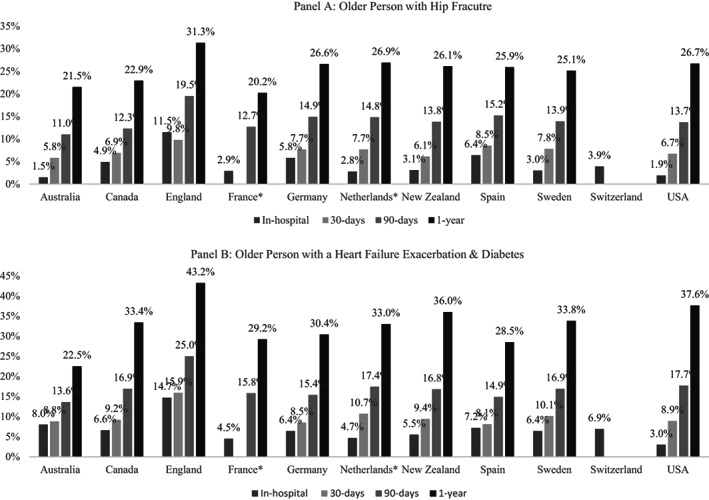
Mortality rates across countries. *Not able to identify the exact date of death outside the hospital. France was only able to identify the quarter of death and, therefore, does not report 30‐day overall mortality and may have slightly upward‐biased estimates for the other mortality estimates, aside from in‐hospital mortality. In the Netherlands, deaths were registered on the last date of each month. Therefore, an additional time window of 15 days was used calculate mortality rates

Figure 1 shows in‐hospital mortality and overall mortality (at 30, 90, and 365 days) for the hip fracture (1a) and heart failure (1b) personas. We observed considerable variation across countries. Australia reported the lowest in‐hospital mortality (1.5%) for the hip fracture patient, whereas England reported the highest in‐hospital mortality at 11.5%. The average in‐hospital mortality across the group of countries was 4.1%. For CHF with diabetes, the average in‐hospital mortality across countries was 6.3%, with the United States reporting lowest in‐hospital mortality at 3.0% and England the highest at 14.7%.

Most countries were able to provide estimates for 30‐day overall mortality for both personas, with the exception of France and Switzerland. For hip fracture, the average mortality across the nine countries able to provide data was 7.1%, and for CHF with diabetes, it was slightly higher at 9.6%. For the hip fracture persona, Australia reported the lowest 30‐day mortality rate at 5.5%, while Spain reported the lowest rate for the CHF with diabetes persona at 8.1%. Across both personas, England reported the highest (9.8% for hip fracture and 15.9% for CHF with diabetes). Notably, the English rate of 30‐day overall mortality for hip fracture was lower in‐hospital mortality (measured at any time).

All countries, except for Switzerland, were able to provide estimates of 90‐ and 365‐day mortality. On average, 90‐day mortality for the hip fracture persona and CHF with diabetes persona was 14.0% and 16.9%, respectively, across all countries. England reported the highest mortality for both types of patients, while Australia reported the lowest for both patients. Finally, at the year mark, the mean mortality across all countries was 25.3% for the hip fracture persona and 32.7% for CHF with diabetes persona. England again reported the highest mortality for both personas, at 31.3% for hip fracture and 43.2% for CHF with diabetes. The US reported the second highest mortality rates (hip fracture at 26.7%; CHF with diabetes at 37.6%). Canada, Australia, and France had the lowest 365‐day mortality for hip fracture at 22.9%, 21.5%, and 20.2%, respectively. France, Spain, and Australia had the lowest 365‐day mortality for CHF with diabetes at 29.2%, 28.5%, and 22.5%, respectively.

### 3.3 Relationship of mortality rates with comorbidities

**FIGURE 2:**
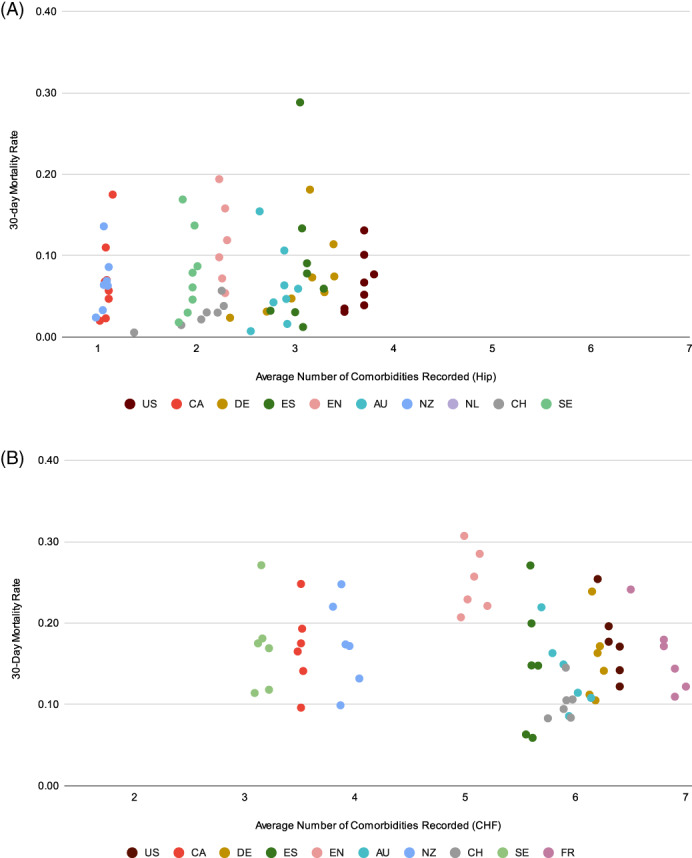
(A) Relationship of comorbidity to 30‐day mortality by 5‐year age groups, hip fracture persona. US = the United States, CA = Canada, DE = Germany, ES=Spain, EN = England, AU = Australia, NZ = New Zealand, NL = the Netherlands, CH = Switzerland, SE = Sweden. (B) Relationship of comorbidity to 30‐day mortality by 5‐year age groups, heart failure and diabetes persona. US = the United States, CA = Canada, DE = Germany, ES = Spain, EN = England, AU = Australia, NZ = New Zealand, NL = the Netherlands, CH = Switzerland, SE = Sweden, FR = France [Color figure can be viewed at wileyonlinelibrary.com]

We examined the correlation between mortality rates to mean number of comorbidities recorded in each country (Figure 2A,B). We observed that within countries, the average number of comorbidities recorded for patients of different age groups changes very little, despite mortality rates showing a clear relationship to the different age categories, where patients of different age had different mortality.

### 3.4 Relationship of in‐hospital mortality with index LOS

We also examined the association of in‐hospital mortality with index inpatient LOS (Figures S1a, S1b). For hip fracture, there is a positive relationship between the two variables, where longer LOS is associated with greater inpatient mortality (Pearson's *r* = 0.72, *p* = 0.00). While still positive, there is a weaker association between in‐hospital mortality and index inpatient LOS for the CHF persona (Pearson's *r* = 0.20, *p* = 0.11).

### 3.5 Readmission rates across countries

**FIGURE 3:**
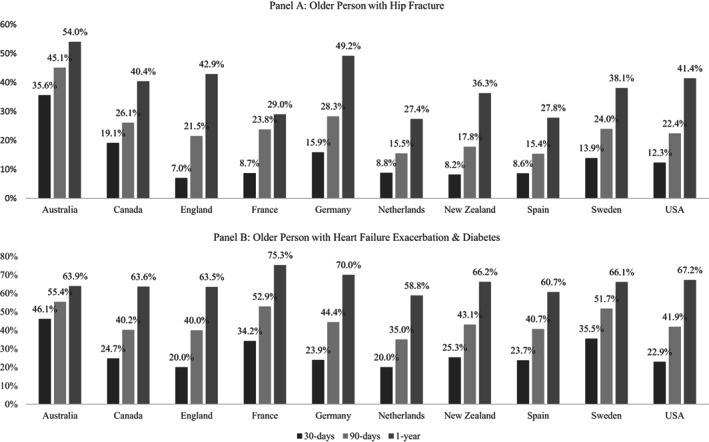
Hospital readmission rates

Figure 3 shows readmission rates at 30, 90, and 365 days. Readmission rates for all periods were higher for the CHF with diabetes persona than the hip fracture persona. At 30 days, the average readmission rate for the hip fracture persona was 13.8% and 27.6% for the CHF with diabetes persona. At 30 days, Australia had notably high readmission rates for both personas, while England had the lowest readmission rates for both personas.

At 90 and 365 days, Australia continued to have the highest readmission rates relative to other countries for the hip fracture persona, followed closely by Germany and England at 365 days. For the CHF persona, France and Germany have the highest 1‐year rates (75.3% and 70.0%, respectively), and the Netherlands had the lowest rate at 58.8%.

### 3.6 Relationship of mortality and readmissions

Finally, we examined the relationship between in‐hospital mortality and 30‐day readmissions. We observed a low negative correlation between them for both personas (hip: Pearson's *r* = −0.34, *p* = 0.00, CHF with diabetes: Pearson's *r* = −0.13, *p* = 0.27).

### 3.7 Time to surgery for hip fracture

**FIGURE 4:**
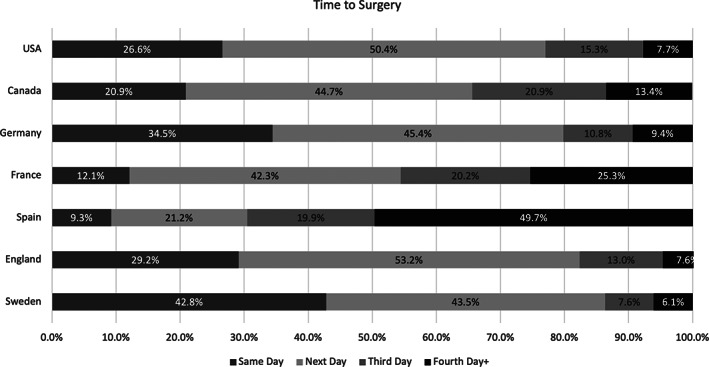
Time to surgery (hip fracture persona)

As a measure of quality of care for the hip fracture persona, seven countries were able to estimate the “time to surgery” (Figure 4). Across all countries, apart from Spain, over 50% of the sample was receiving surgery either on the same day or the day following admission. In Sweden, this was over 85% of the sample. In England and the United States, the rate of same day or next day surgery exceeded 75%. Spain (Aragon), France, and Canada (Ontario) had the lowest proportion of patients receiving surgery the same day (9.3%, 12.1%, and 20.9%, respectively).

## 4 DISCUSSION

In this study, we examined key health system outcomes for two HNHC personas—an older frail adult with a hip fracture and an older adult with heart failure and diabetes—across 11 high‐income countries. Our approach builds on existing comparative work by exploring mortality and readmissions for two complex patients for which there is limited comparable data across countries. We found substantial variation in mortality and readmission rates across countries, for both personas. For the hip fracture persona, we also found differences in time to surgery from day of admission to hospital. Our results have important implications for the research community interested in conducting international comparisons as well as national policy makers interested in understanding their own relative performance in caring for complex personas. We highlighted some key findings that emerge from these results.

First, we found variation in mortality rates for both personas across countries at different time intervals. The notable exception to this is England, which consistently reports the highest mortality rates for both personas across all time intervals. Prior work has found lower 30‐day mortality rates for hip fracture patients in England (by around 3 percentage points) using data reported by the national hip fracture registry for the same time period,^14^ which would still place England among the countries with the highest mortality rate. Another study similarly reported lower mortality at 365 days.^15^ However, both of these other studies included patients younger than age 65 in their sample, which may influence their mortality estimates. Furthermore, the English data used in this study reflect mortality across a range of years starting from 2014 and may not adequately capture substantial improvements that have been made in recently. More work should be carried out on more recent data from a larger national sample to validate these findings. As time to surgery was consistent with other countries, further research is needed to investigate the role of rehab and postacute care (delivered by community care and social care, which are separately organized and funded from the National Health Service) to understand if this is contributing to the longer inpatient LOS and higher mortality rates.

Second, after England, we found that the United States ranks the second highest for 365‐year mortality across both personas. This is notable, as the United States spends comparatively more resources caring for these patients^16^, ^17^ and suggests that they are the least efficient system. Unlike England, the United States has slightly lower average 30‐day mortality for both personas. While related analyses have shown that the United States is spending more both because of a comparatively higher unit cost and more utilization of postacute care,^16^, ^17^ these results suggest that this increased resource use may not be translating into improved long‐term outcomes. Prior work has similarly found that the United States performs above average when it comes to measuring performance related to the period immediately following a hospitalization but then lags behind other countries when assessing longer, population‐level health outcomes.^18^ The US policy makers should further investigate how much spending is relatively allocated to health care services for acute and postacute care services versus long‐term care services, which are currently not covered for the majority of Medicare patients (such as those who are not also covered by Medicaid), to better understand the impact this may have on patient outcomes. Other countries, like the Netherlands and Canada, two of the countries with the lowest rate of death at 365 days, have more comprehensive long‐term care services support for older adults.^19^, ^20^

Third, as has been noted elsewhere, HNHC patients are heterogeneous groups, which require different care.^21^ To date, there has been limited international work examining more homogenous subtypes to see if the same countries consistently perform well in treating different types of patients. We found some variability in relative performance of countries across the two personas, suggesting that the design of different systems may influence the final outcomes of these patients, particularly where specific policies have been implemented to improve coordination of care. For example, Spain has relatively low mortality for the CHF persona, it fares worse relative to other countries on short‐term mortality for hip fracture, possibly related to the longer delays in receiving surgery after being admitted to the hospital,^22^, ^23^ which should be explored further. To further understand the role health system design and coordination of care influence outcomes, policy makers should focus on making more data available that follows patients across care settings. This can be useful to improve the rigor of comparative analysis, such as this one, as well as national research on this topic.

Fourth, we also explore the relationship between the number of recorded comorbidities for patients to 30‐day mortality. We observed variability in the average number of comorbidities recorded across countries, which appear to have more to do with local coding practices rather than the severity of the patients, as illustrated by the relative similarity in the number of comorbidities coded by age group within countries.

Different incentives across countries may influence the coding of secondary diagnoses and has also been observed elsewhere.^24^ For example, in the United States and Germany, insurance systems where reimbursement is related to patient severity, we see much greater levels of comorbidity recorded than countries like Canada, Sweden, and England where providers are reimbursed through global budgets. As a result, adjusting mortality by the number of comorbidities across countries may introduce bias.

Fifth, we found substantial variation in readmission rates for both personas across countries. This is likely influenced by several factors, including differences in the amount of time patients spend in the hospital and differential use of postacute care rehabilitative services or long‐term care.^16^, ^17^ Unfortunately, the majority of countries lack comprehensive data on long‐term care and postacute rehabilitative care, limiting our ability to explore these relationships further. Of note, in‐hospital mortality was weakly inversely associated with readmission rate, suggesting a problem of competing risk. In other words, if more patients are dying in the index hospitalization in one country, then a comparatively healthier population is being discharged and serves as the denominator for readmission as has been shown in other work.^25^, ^26^ Our work suggests that in order to better interpret cross‐national variation in readmission rate, more comprehensive data on patient trajectories need to be collected and made available to researchers.

Finally, we observed important differences in aspects of quality of care for the hip patients, namely time to surgery, for a subset of countries. Time to surgery for hip fracture patients has been shown to influence outcomes, including mortality.^27^, ^28^ We found considerable variability across countries in this metric. Sweden and England have high proportion of patients receiving surgery by the next day possibly because this target has been recognized in guidelines.^29^ More variables detailing important clinical processes are necessary to better identify actionable areas of improvement across countries and bring together a more complete picture of variations in care.

This paper makes an important contribution to the literature on performance comparisons of health system. Other studies and reports have explored the overall performance of the United States to other high‐income health systems.^18^ To our knowledge, this is one of the first articles that provide detailed international data comparing the health outcomes of two HNHC personas as follows: older patients with hip fracture and a multimorbid patient with heart failure and diabetes. Other comparative studies have explored outcomes for specific patient populations, including 30 day and year‐long mortality, and cost‐quality, for other common procedures, such as AMI, stroke, and elective hip replacement.^5^, ^6^, ^30^, ^31^, ^32^, ^33^ In addition, 30‐day case fatality rates for AMI and stroke are also reported by the OECD health care quality indicators project (https://www.oecd.org/els/health-systems/hcqi-acute-care.htm). We are not aware of other work that comparing hospital readmission rates across more than three countries.^34^

### 4.1 Limitations

There are several limitations to this work. First, while we have carried out our best to ensure data comparability across countries, there are some differences in national coding practices and the representativeness of the data in certain countries that in turn may influence the results. We noted particular issues with the comparability of the mortality estimates from the French and Dutch data, which may lead to an underestimation given that they can only record overall deaths at fixed time periods. In addition, we do not adjust for comorbidities given the apparent differences in coding practices across countries. However, given that all patients have similar primary diagnosis and we adjust for demographics, we did not believe much additional variability would be accounted for by this adjustment. Second, we were not able to adjust for a number of factors that are important predictors of both short‐ and long‐term mortality, such as baseline severity of the clinical diagnoses of heart failure or diabetes, nor are we able to account for differences in socioeconomic status. Third, while we do report a set of comparative outcomes across countries, we are missing some of the most important outcomes for these populations, including mobility outcomes following hip fracture, time spent at home, and patient‐reported outcomes. Further data linkage to all sectors, particularly postacute and long‐term care, is needed to make this possible across countries. Fourth, while this article, and the accompanying body of work, produces detailed data outlining differences in the utilization, expenditure, and outcomes of care for these two patient personas, this work is descriptive, and we cannot make any causal inferences about which factors in each system impact the observed outcomes. However, these results can serve to point to areas that merit further investigation. Finally, due to data restrictions across countries, we were unable to pool data together to conduct more rigorous analyses of the patient‐level data. In order to advance comparative health systems research in the future and provide more detailed advice to national policy makers, it is important that suitable workarounds to this issue are found, while still protecting the privacy of the data.

## 5 CONCLUSIONS

Taken together, these findings suggest that there are meaningful differences in health system outcomes across the 11 countries studied, for two types of HNHC patients as follows: an older frail person with hip fracture and patients with CHF with diabetes. Further efforts to link in additional data, particularly on long‐term care, would be beneficial to further understand variability in health outcomes and health system efficiency.

## Supporting information


**Table S1.** Patient characteristics of hip fracture persona
**Table S2.** Patient characteristics of the heart failure persona with diabetes
**Figure S1a.** Relationship of index inpatient length of stay to inpatient mortality by 5‐year age groups, hip
**Figure S1b.** Relationship of index inpatient length of stay to inpatient mortality by 5‐year age groups, congestive heart failure (CHF) +diabetes mellitus (DM)
**Figure S2a.** Relationship of readmissions and inpatient mortality by 5‐year age groups, hip persona
**Figure S2b.** Relationship of readmissions and inpatient mortality by 5‐year age groups, congestive heart failure (CHF) + diabetes mellitus (DM) persona
