# Supplementary material for: Differences in health outcomes for high‐need high‐cost patients across high‐income countries
Source: Health Serv Res. 2021 Aug 11;56(Suppl 3):1347–57. doi: 10.1111/1475-6773.13735 (PMC8579207; doi:10.1111/1475-6773.13735)
Supplement: Supplementary file 1 — Table S1. Patient characteristics of hip fracture persona Table S2. Patient characteristics of the heart failure persona with diabetes Figure S1a. Relationship of index inpatient length of stay to inpatient mortality by 5‐year age groups, hip Figure S1b. Relationship of index inpatient length of stay to inpatient mortality by 5‐year age groups, congestive heart failure (CHF) +diabetes mellitus (DM) Figure S2a. Relationship of readmissions and inpatient mortality by 5‐year age groups, hip persona Figure S2b. Relationship of readmissions and inpatient mortality by 5‐year age groups, congestive heart failure (CHF) + diabetes mellitus (DM) persona [file HESR-56-1347-s001.docx]

**Appendix**

**Table of Contents:**

**1. Appendix Table 1.** Patient Characteristics of Hip Fracture Persona

**2. Appendix Table 2.** Patient Characteristics of the Heart Failure Persona with Diabetes

**3. Appendix Figure 1a.** Relationship of Index Inpatient Length of Stay to Inpatient Mortality by 5-year age groups, Hip

**4. Appendix Figure 1b.** Relationship of Index Inpatient Length of Stay to Inpatient Mortality by 5-year age groups, CHF+DM

**5. Appendix Figure 2a.** Relationship of Readmissions and Inpatient Mortality by 5-year age groups, Hip Persona

**6. Appendix Figure 2b.** Relationship of Readmissions and Inpatient Mortality by 5-year age groups, CHF+DM Persona

**Appendix Table 1: Patient Characteristics of Hip Fracture Persona**

| **Patient Characteristics** | **Australia** | **Canada** | **England** | **France** | **Germany** | **Netherlands** | **New Zealand** | **Spain** | **Sweden** | **Switzerland** | **USA** |
| --- | --- | --- | --- | --- | --- | --- | --- | --- | --- | --- | --- |
| **Total No. of Patients** | 2,511 | 9,872 | 2,738 | 42,849 | 13,998 | 4,463 | 2,940 | 1,859 | 14,764 | 6,860 | 29,134 |
| **Age** |  |  |  |  |  |  |  |  |  |  |  |
| mean (+/- standard deviation) | 84.7  (7.7) | 83.4  (8.1) | 83.5  (7.9) | 84.3  (7.7) | 83.5  (7.7) | 82.2  (8.0) | 84.0  (7.8) | 85.4  (7.0) | 83.2  (7.6) | 81.2  (6.9) | 83.2  (8.3) |
| median | 86 | 85 | 84 | 85 | 84 | 83 | 85 | 84 | 84 | 82 | 84 |
| **Sex** |  |  |  |  |  |  |  |  |  |  |  |
| Male | 37.2% | 29.4% | 29.0% | 22.9% | 24.1% | 29.2% | 29.6% | 23.3% | 32.4% | 26% | 28.6% |
| Female | 62.8% | 70.7% | 71.0% | 77.1% | 75.9% | 70.8% | 70.4% | 76.7% | 67.6% | 73.7% | 71.4% |
| **Chronic Conditions (Elixhauser)** |  |  |  |  |  |  |  |  |  |  |  |
| Congestive Heart Failure | 12.4% | 6.0% | 10.9% | 8.3% | 21.6% | n/a | 4.7% | 8.1% | 11.0% | 8.4% | 17.30% |
| Cardiac Arrhythmia | 29.2% | 15.1% | 27.1% | 21.9% | 28.7% | n/a | 13.7% | 18.1% | 22.1% | 20.6% | 33.9% |
| Valvular Disease | 4.1% | 1.6% | 7.3% | 3.8% | 7.9% | n/a | 1.6% | 6.0% | 3.2% | 5.5% | 11.8% |
| Pulmonary Circulation Disorders | 4.2% | 1.2% | 2.1% | 1.0% | 3.0% | n/a | 1.0% | 1.9% | 0.8% | 2.5% | 0.8% |
| Peripheral Vascular Disorders | 2.6% | 0.8% | 3.8% | 2.7% | 6.9% | n/a | 0.5% | 2.0% | 1.4% | 4.5% | 7.6% |
| Hypertension (uncomplicated or complicated) | 24.9% | 25.9% | 55.0% | 41.1% | 71.3% | n/a | 11.6% | 58.5% | 41.3% | 51.6% | 77.1% |
| Paralysis | 4.1% | 0.4% | 1.6% | 1.8% | 4.4% | n/a | 1.9% | 1.6% | 1.5% | 2.0% | 3.4% |
| Other Neurological Disorders | 8.5% | 3.4% | 7.4% | 2.8% | 10.0% | n/a | 3.7% | 14.8% | 5.0% | 7.6% | 20.5% |
| Chronic Pulmonary Disease | 7.3% | 6.3% | 22.0% | 5.0% | 10.1% | n/a | 4.0% | 8.0% | 9.5% | 8.1% | 22.2% |
| Diabetes (uncomplicated or complicated) | 17.6% | 19.5% | 15.1% | 12.4% | 19.8% | n/a | 15.0% | 20.2% | 14.2% | 14.2% | 22.5% |
| Hypothyroidism | 1.3% | 2.1% | 10.3% | 6.1% | 17.4% | n/a | 0.3% | 3.6% | 5.5% | 7.3% | 23.9% |
| Renal Failure | 12.2% | 4.0% | 15.2% | 6.3% | 26.9% | n/a | 8.3% | 9.0% | 5.0% | 19.4% | 19.2% |
| Liver Disease | 1.2% | 0.5% | 1.0% | 0.7% | 1.8% | n/a | *suppressed | 1.2% | 0.6% | 1.1% | 1.5% |
| Peptic Ulcer Disease excluding bleeding | 0.4% | 0.20% | 0.6% | 0.1% | 0.2% | n/a | *suppressed | 0.0% | 0.1% | 0.2% | 0.7% |
| AIDS/HIV | 0.0% | 0.0% | *suppressed | 0.0% | 0.0% | n/a | *suppressed | 0.0% | 0.0% | 0.0% | 0.0% |
| Lymphoma | 0.6% | 0.4% | *suppressed | 0.4% | 0.3% | n/a | *suppressed | 0.3% | 0.5% | 0.4% | 0.6% |
| Metastatic Cancer | 1.9% | 1.1% | 1.9% | 0.9% | 0.9% | n/a | 0.5% | 0.9% | 1.1% | 1.1% | 1.1% |
| Solid Tumor without Metastasis | 3.2% | 2.3% | 3.4% | 0.8% | 2.5% | n/a | 0.7% | 2.2% | 4.0% | 2.5% | 2.0% |
| Rheumatoid Arthritis/collagen | 1.4% | 0.7% | 6.0% | 1.1% | 2.4% | n/a | 0.2% | 1.6% | 3.4% | 2.0% | 4.2% |
| Coagulopathy | 6.6% | 1.7% | 0.8% | 1.3% | 7.5% | n/a | 2.8% | 0.7% | 0.4% | 6.3% | 8.4% |
| Obesity | 0.6% | 0.4% | 1.1% | 2.7% | 3.5% | n/a | *suppressed | 2.2% | 0.2% | 0.8% | 4.3% |
| Weight Loss | 20.3% | 0.5% | 1.0% | 19.3% | 10.7% | n/a | 1.6% | 2.9% | 0.4% | 8.9% | 7.1% |
| Fluid and Electrolyte Disorders | 38.0% | 10.3% | 15.3% | 12.7% | 35.8% | n/a | 28.9% | 3.1% | 1.9% | 17.3% | 31.0% |
| Blood Loss Anemia | 3.0% | 0.2% | *suppressed | 2.7% | 1.0% | n/a | 0.6% | 0.3% | 0.1% | 1.3% | 2.3% |
| Deficiency Anemia | 5.4% | 1.1% | 3.0% | 6.8% | 4.1% | n/a | 1.6% | 10.0% | 1.0% | 3.0% | 23.4% |
| Alcohol Abuse | 1.7% | 1.3% | 2.9% | 1.8% | 2.0% | n/a | 1.5% | 0.5% | 1.2% | 3.1% | 2.5% |
| Drug Abuse | 0.4% | 0.1% | *suppressed | 1.1% | 0.7% | n/a | *suppressed | 0.1% | - | 0.9% | 0.6% |
| Psychoses | 0.2% | 0.4% | 0.4% | 0.7% | 1.0% | n/a | *suppressed | 1.0% | 0.7% | 1.0% | 2.1% |
| Depression | 3.9% | 1.5% | 6.9% | 5.8% | 11.0% | n/a | 0.2% | 8.3% | 2.0% | 8.4% | 15.3% |
| **No. of chronic conditions (mean, median, S.D.)** |  |  |  |  |  |  |  |  |  |  |  |
| mean (+/- standard deviation) | 2.9 (1.9) | 1.1 (1.2) | 2.2 (1.5) | 1.7 (1.5) | 3.2 (2.1) | n/a | 1.1 (1.2) | 3.1 (1.5) | 2.0 (1.1) | 2.1 (1.8) | 3.7 (2.0) |
| median | 2 | 1 | 2 | 1 | 3 |  | 1 | 3 | 2 | 2 | 3 |

**Notes:** *Values are suppressed by the data vendor due to low volumes of patients

**Appendix Table 2. Patient Characteristics of the Heart Failure Persona with Diabetes**

| **Patient Characteristics** | **Australia** | **Canada** | **England** | **France** | **Germany** | **Netherlands** | **New Zealand** | **Spain** | **Sweden** | **Switzerland** | **USA** |
| --- | --- | --- | --- | --- | --- | --- | --- | --- | --- | --- | --- |
| **Total No. of Patients** | 3,014 | 6,305 | 742 | 21,957 | 10,583 | 2,035 | 1,572 | 1,270 | 4,615 | 3,369 | 21,803 |
| **Age** |  |  |  |  |  |  |  |  |  |  |  |
| mean (+/- standard deviation) | 79.4  (6.8) | 78.1 (7.0) | 78.7 (6.6) | 79.1 (6.9) | 79.0 (6.4) | 76.2 (5.6) | 77.3 (6.9) | 80.2 (5.1) | 80.3 (6.8) | 78.6 (6.5) | 77.2 (7.0) |
| median | 80 | 79 | 79 | 80 | 79 | 77 | 77 | 81 | 81 | 77 | 77 |
| **Sex** |  |  |  |  |  |  |  |  |  |  |  |
| Male | 63.5% | 54.6% | 57.5% | 54.0% | 49.4% | 51.6% | 57.6% | 53.1% | 58.7% | 57.4% | 50.0% |
| Female | 36.5% | 45.4% | 42.5% | 46.0% | 50.6% | 48.4% | 42.4% | 46.9% | 41.3% | 42.6% | 50.0% |
| **Chronic Conditions (Elixhauser)** |  |  |  |  |  |  |  |  |  |  |  |
| Congestive Heart Failure | 100.0% | 100.0% | 100.0% | 100.0% | 100.0% | n/a | 100.0% | 100.0% | 100.0% | 100.0% | 100.0% |
| Cardiac Arrhythmia | 54.6% | 32.8% | 59.7% | 64.9% | 68.1% | n/a | 27.7% | 62.4% | 61.6% | 62.6% | 58.8% |
| Valvular Disease | 15.2% | 9.0% | 26.8% | 27.2% | 31.4% | n/a | 8.8% | 33.5% | 12.5% | 30.5% | 0.3% |
| Pulmonary Circulation Disorders | 13.4% | 4.6% | 7.6% | 13.8% | 17.5% | n/a | 6.7% | 19.4% | 3.7% | 14.9% | 0.0% |
| Peripheral Vascular Disorders | 7.6% | 2.0% | 8.8% | 14.9% | 17.3% | n/a | 1.7% | 8.5% | 4.2% | 18.2% | 15.1% |
| Hypertension (uncomplicated or complicated) | 51.6% | 41.1% | 64.2% | 69.0% | 82.4% | n/a | 51.2% | 75.7% | 57.2% | 81.2% | 89.3% |
| Paralysis | 3.6% | 0.2% | 1.4% | 1.9% | 3.2% | n/a | 1.2% | 2.0% | 1.3% | 1.7% | 2.9% |
| Other Neurological Disorders | 4.7% | 0.9% | 3.2% | 2.2% | 5.1% | n/a | 1.2% | 7.2% | 2.1% | 3.4% | 9.0% |
| Chronic Pulmonary Disease | 26.2% | 16.3% | 31.0% | 17.1% | 22.7% | n/a | 10.3% | 24.6% | 18.7% | 17.7% | 42.7% |
| Diabetes (uncomplicated or complicated) | 100.0% | 99.9% | 100.0% | 100.0% | 99.7% | n/a | 100.0% | 100.0% | 100.0% | 100.0% | 100.0% |
| Hypothyroidism | 1.7% | 2.1% | 10.0% | 9.0% | 16.8% | n/a | 0.6% | 8.4% | 5.2% | 11.1% | 21.8% |
| Renal Failure | 49.3% | 5.5% | 39.2% | 34.9% | 59.0% | n/a | 47.3% | 42.9% | 25.8% | 62.1% | 54.5% |
| Liver Disease | 4.3% | 1.8% | 3.6% | 3.6% | 4.3% | n/a | 1.2% | 3.9% | 1.3% | 3.2% | 3.3% |
| Peptic Ulcer Disease excluding bleeding | 0.5% | * | * | 0.3% | 0.1% | n/a | * | 0.1% | 0.2% | 0.4% | 0.7% |
| AIDS/HIV | 0.0% | 0.0% | * | 0.1% | 0.0% | n/a | * | 0.0% | * | 0.1% | 0.0% |
| Lymphoma | 1.2% | 0.6% | * | 0.8% | 0.3% | n/a | 0.4% | 0.4% | 0.7% | 0.8% | 0.9% |
| Metastatic Cancer | 3.1% | 0.7% | * | 1.3% | 0.9% | n/a | 1.2% | 0.9% | 0.7% | 1.7% | 1.1% |
| Solid Tumor without Metastasis | 5.5% | 2.5% | 4.2% | 1.5% | 3.1% | n/a | 1.5% | 3.4% | 3.9% | 4.0% | 2.2% |
| Rheumatoid Arthritis/collagen | 1.7% | 0.4% | 5.4% | 1.2% | 2.5% | n/a | * | 1.9% | 3.8% | 2.4% | 3.0% |
| Coagulopathy | 13.9% | 3.1% | * | 2.6% | 5.4% | n/a | 3.8% | 2.4% | 0.5% | 8.1% | 6.3% |
| Obesity | 6.5% | 3.2% | 9.6% | 23.2% | 18.0% | n/a | 2.7% | 19.1% | 4.2% | 7.3% | 28.1% |
| Weight Loss | 11.9% | 0.7% | * | 15.2% | 3.0% | n/a | 0.6% | 3.9% | 0.2% | 5.6% | 4.2% |
| Fluid and Electrolyte Disorders | 49.4% | 16.7% | 19.1% | 24.4% | 35.0% | n/a | 16.8% | 8.2% | 2.6% | 27.4% | 34.5% |
| Blood Loss Anemia | 4.2% | 0.3% | * | 2.1% | 1.0% | n/a | * | 2.7% | 0.2% | 2.3% | 1.2% |
| Deficiency Anemia | 11.9% | 3.3% | 5.7% | 9.6% | 6.0% | n/a | 5.2% | 25.6% | 3.1% | 11.0% | 36.5% |
| Alcohol Abuse | 2.5% | 0.8% | * | 3.5% | 1.0% | n/a | 1.0% | 1.3% | 0.9% | 2.7% | 1.2% |
| Drug Abuse | 0.3% | 0.0% | * | 0.1% | 0.0% | n/a | * | 0.0% | * | 0.7% | 0.5% |
| Psychoses | 0.4% | 0.2% | * | 0.5% | 0.0% | n/a | * | 0.2% | 0.5% | 0.5% | 1.8% |
| Depression | 4.0% | 1.5% | 3.5% | 4.7% | 6.3% | n/a | 0.4% | 6.6% | 1.0% | 8.0% | 1.5% |
| **No. of chronic conditions (mean, median, S.D.)** |  |  |  |  |  |  |  |  |  |  |  |
| mean (+/- standard deviation) | 5.9 (2.3) | 3.5 (1.3) | 5.1 (1.5) | 5.5 (1.9) | 6.1 (2.0) | n/a | 3.9 (1.4) | 5.6 (2.1) | 3.2 (1.2) | 5.9 (1.7) | 6.3 (1.7) |
| median | 6 | 3 | 5 | 5 | 6 | n/a | 4 | 5 | 3 | 6 | 6 |

**Notes:** *Values are suppressed due to low volumes of patients

**Appendix Figure 1a: Relationship of Index Inpatient Length of Stay to Inpatient Mortality by 5-year age groups, Hip**

r = 0.74

*Legend: US=United States, CA=Canada, DE=Germany, ES=Spain, EN=England, AU=Australia, NZ=New Zealand, NL=Netherlands, CH=Switzerland, SE=Sweden*

**Appendix Figure 1b: Relationship of Index Inpatient Length of Stay to Inpatient Mortality by 5-year age groups, CHF+DM**

*****Legend: US=United States, CA=Canada, DE=Germany, ES=Spain, EN=England, AU=Australia, NZ=New Zealand, NL=Netherlands, CH=Switzerland, SE=Sweden , FR=France*

r = 0.21

**Appendix Figure 2a: Relationship of Readmissions and Inpatient Mortality by 5-year age groups, Hip**

r = -0.34

*Legend: US=United States, CA=Canada, DE=Germany, ES=Spain, EN=England, AU=Australia, NZ=New Zealand, NL=Netherlands, CH=Switzerland, SE=Sweden*

**Appendix Figure 2b: Relationship of Readmissions and Inpatient Mortality by 5-year age groups, CHF+DM**

*****Legend: US=United States, CA=Canada, DE=Germany, ES=Spain, EN=England, AU=Australia, NZ=New Zealand, NL=Netherlands, CH=Switzerland, SE=Sweden , FR=France*

r = -0.18
